# Mortality Attendant upon Homæopathic Practice in New-York

**Published:** 1846-10

**Authors:** 


*Mortality attendant upon Homœopathic practice in New-York.*—Dr. Cox in a communication for the N. York Medical and Surgical Reporter, stated that the practitioner who certified to the largest number of deaths was a homœopathist. This a homœopathic writer denied in an article published in the Tribune. Whereupon the editor of the Reporter publishes a written certificate from a health inspector to that effect.

